# Can a robot laugh with you?: Shared laughter generation for empathetic spoken dialogue

**DOI:** 10.3389/frobt.2022.933261

**Published:** 2022-09-15

**Authors:** Koji Inoue, Divesh Lala, Tatsuya Kawahara

**Affiliations:** Graduate School of Informatics, Kyoto University, Kyoto, Japan

**Keywords:** laughter generation, shared laughter, empathy, spoken dialogue system, android robot, laughter type

## Abstract

Spoken dialogue systems must be able to express empathy to achieve natural interaction with human users. However, laughter generation requires a high level of dialogue understanding. Thus, implementing laughter in existing systems, such as in conversational robots, has been challenging. As a first step toward solving this problem, rather than generating laughter from user dialogue, we focus on “shared laughter,” where a user laughs using either solo or speech laughs (initial laugh), and the system laughs in turn (response laugh). The proposed system consists of three models: 1) initial laugh detection, 2) shared laughter prediction, and 3) laugh type selection. We trained each model using a human-robot speed dating dialogue corpus. For the first model, a recurrent neural network was applied, and the detection performance achieved an F1 score of 82.6%. The second model used the acoustic and prosodic features of the initial laugh and achieved a prediction accuracy above that of the random prediction. The third model selects the type of system’s response laugh as social or mirthful laugh based on the same features of the initial laugh. We then implemented the full shared laughter generation system in an attentive listening dialogue system and conducted a dialogue listening experiment. The proposed system improved the impression of the dialogue system such as empathy perception compared to a naive baseline without laughter and a reactive system that always responded with only social laughs. We propose that our system can be used for situated robot interaction and also emphasize the need for integrating proper empathetic laughs into conversational robots and agents.

## 1 Introduction

Dialogue systems are commonly implemented in robots and virtual agents, with applications in task-based and conversational scenarios. For conversational scenarios, the focus is on natural language processing and on the emulation of other real conversational phenomena, such as backchannels, turn-taking, and fillers (Inoue et al., 2016; Hara et al., 2018; Hussain et al., 2019; Lala et al., 2019; Skantze, 2021). Laughter is another such phenomenon.

The implementation of a laughter model is a non-trivial task. Systems that try to emulate everyday conversation still struggle with the notion of when to laugh. Laughter stimuli may not be explicit, although humor recognition in a textual medium can produce reasonable results (Chen and Soo, 2018; Weller and Seppi, 2019; Annamoradnejad and Zoghi, 2020). Situated conversation presents issues such as incorrect speech recognition, prosody, and timing that may complicate a system’s ability to respond adequately to a joke in real-time. Furthermore, the type of laughter used as a reaction to a stimulus can influence the atmosphere of a conversation. For example, a user who describes an unfortunate event they experienced may be satisfied with a sympathetic chuckle, but a cheerful laugh would be inappropriate and could make the user feel embarrassed.

Given these types of issues in situated conversation, we propose another method of laughter implementation, *shared laughter*, in which the user initially laughs, and then the system responds with laughter as an empathetic response. In the case of human–human behavior, research has suggested that shared laughter can be framed in terms of speaker invitation and listener acceptance (Glenn, 1991). Furthermore, it is clear that not all speaker laughs are invitations to respond with a shared laugh, so humans have to decide when it is appropriate to respond with laughter (Holt, 2010; Bonin et al., 2014). The prosodic and acoustic features of the laughter itself also differ between initial and response laughs (Truong and Trouvain, 2014). Therefore, shared laughter in the real world cannot be reduced to a call-and-response mechanism. This motivates us to construct a more appropriate computational model for shared laughter.

**FIGURE 1:**
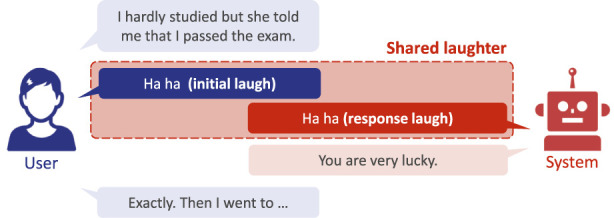
Example of shared laughter by spoken dialogue systems.

Figure 1 shows an overview of the proposed system behavior designed to emulate human shared laughter. Similar to backchanneling (Yalçın and DiPaola, 2019), shared laughter affects the emotional response of the participant (Neves et al., 2018), but importantly, it does not require the system to completely understand user dialogue or the conversation thread. In real-world applications, we can imagine an analogous situation in which a foreign language is not understood. If a speaker of that language suddenly laughed, we may feel inclined to laugh with them, although we are unaware of the context, and this would be an inappropriate reaction in some cases. We propose that engaging in shared laughter at critical times can improve the perceived empathy of the system and should be a requirement for conversational robots.

From a systems perspective, the shared laughter approach requires completing several sub-tasks. The first sub-task is laughter detection, which simply determines if a user has laughed. Once a laugh is detected, the next step is to decide if the agent should laugh as a response. Our study demonstrates that this is a less common occurrence than unshared laughter. We also propose that the type of laughter generated by the system should be considered. Although humans have a vast range of laughs, a system may be restricted to only a few fixed laugh utterances. We show that more subtle “social” laughs are a necessary system feature, in addition to more explicit “mirthful” laughs.

The basis of our work is to implement this type of model in a real-time system. Given a user’s utterance, we should be able to detect if the utterance is a laugh, predict if the user should engage in shared laughter, and finally predict the type of laugh that should be used as a reaction.

This study analyzes and annotates a large corpus of human–robot interactions to understand the frequency and types of laughter used in shared laughter. We then extract training data to create models that can address the three tasks described earlier. Finally, we conducted a subjective experiment to evaluate the implemented model in a listening task. This work uses Japanese as the target language with the goal of implementing the shared laughter model in the android ERICA (Inoue et al., 2016).

Our contributions are useful for researchers in conversational dialogue systems. We show that shared laughter improves the perception of the system and that the type of laughter is influential. The sub-tasks of the shared laughter system can be modularized to enable incremental progress. The current implementation is achieved using only audio data and can function in real-time, making it relatively generalizable for other conversation systems.

The remainder of this article is organized as follows: Section 2 summarizes related works. Section 3 introduces the dialogue corpus and annotation of shared laughter samples. Section 4 explains the proposed system, each module, and its evaluations. We evaluate the shared laughter dialogue samples generated by the proposed system using crowdsourcing in Section 5 and discuss limitations and future work before concluding.

## 2 Related work

Laughter has been well studied in scientific literature, including an analysis of its function in human conversation and interaction (Provine, 2001; Glenn, 2003). Hearing laughter from others is known to trigger our laughter and be “contagious” (Provine, 1992), for example, when we see and hear laughter on television. In terms of shared laughter in conversation, it is a conversational behavior and expression that arises as a form of mimicry (Estow et al., 2007; Navarretta, 2016). Furthermore, shared laughter has been the focus of conversation analysis in which the intensity, timing, and the type of response laugh have been systematically studied to identify patterns in laughter behavior (Bonin et al., 2014; Gupta et al., 2015; El Haddad et al., 2019). One study has examined laughter in the context of human–robot interactions (Batliner et al., 2019).

From an intelligent systems perspective, creating models that do automatic laughter detection is arguably the most common task using both audio and visual features for training (Truong and Van Leeuwen, 2007; Cosentino et al., 2016; Turker et al., 2017; Akhtar et al., 2018; Kantharaju et al., 2018; Ataollahi and Suarez, 2019; Gosztolya and Tóth, 2019), and ubiquitous devices such as computer microphones and web cameras can provide reasonably accurate detection. These studies primarily distinguish between speech and laughter for an inter-pausal unit (IPU) or perform detection using a continuous model. They often try to capture information to predict user engagement or other internal states rather than dynamically respond to the actual laugh. Our previous work trained a model to predict shared laughter, although subjective experiments were not conducted, and the method of extracting training samples was not thorough (Lala et al., 2020).

Naturally, researchers have also attempted to integrate laughing behavior into robots and agents. An early example is the AVLaughterCycle (Urbain et al., 2009), which detects laughter from the user and mimics it through the virtual agent Greta. Subsequent studies expanded on this by detecting features of the user’s laughter to generate a more suitable laugh for the agent (Niewiadomski et al., 2013; Hofmann et al., 2015) and increase the engagement and amusement level of the user. Another study used laughter in a social robot during a quiz game to analyze user engagement (Türker et al., 2017). These studies used an external stimulus as a trigger for laughter, such as a funny video or game. Our study targets a different scenario, where the interaction is a dyadic conversation. In this case, we assume that the trigger for laughter is based entirely on the content of the conversation.

Laughter generation in robots and agents has also been addressed but primarily in terms of animation or movement to produce realistic laughs (Niewiadomski and Pelachaud, 2012; Ishi et al., 2016a; Ishi et al., 2019). For the majority of agents, the range of laughter utterances is restricted by the text-to-speech system and is unable to generate speech laughs, although recent research has addressed this issue by producing a large variety of natural-sounding laughs (Mori et al., 2019; Tits et al., 2020; Luong and Yamagishi, 2021).

Our study is positioned as an integrated laughter system for dyadic chatting conversation. We intend the system to be used in a real-time, situated environment and as a module that can be integrated into existing agents and social robot systems instead of as a stand-alone system. We emphasize that the decisions regarding if and how to laugh are a necessary requirement in the laughter system, and current agent systems tend to overlook the importance of laughter type selection.

## 3 Dataset

In this section, we introduce a dataset for analyzing shared laughter and generating samples for model training. We annotated shared laughter samples using a speed dating dialogue corpus.

### 3.1 Speed dating dialogue corpus

**FIGURE 2:**
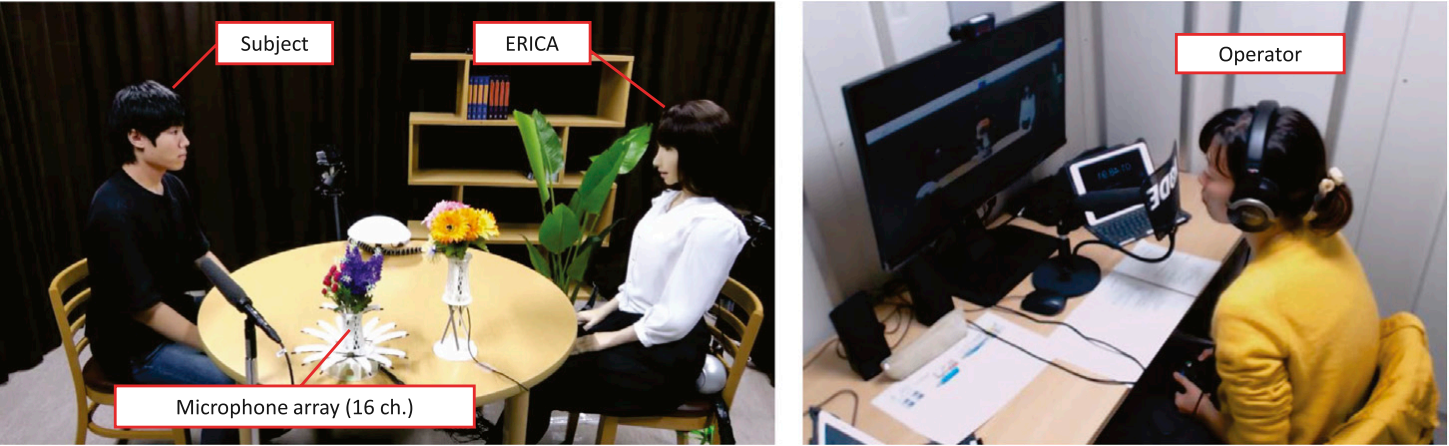
Snapshot of dialogue recording.

The speed dating dialogue corpus contains dialogues between a subject and the teleoperated android ERICA (Inoue et al., 2016) as shown in Figure 2. ERICA’s operator was one of four amateur actresses who was located in another room and directly spoke into a microphone. The uttered speech was then played through ERICA’s speaker. The non-verbal behaviors of ERICA, such as head nodding, eye gaze, and gestures, were controlled by the operator using a controller.

The dialogue task was speed dating, where the purpose of the dialogue is to get to know the other participant. Therefore, participants try to make themselves friendly, and we expect to observe many laughs. The robot (ERICA) is presented as a female, and the subjects were males, recruited from our university.

The duration of each dialogue was 10 to 15 min, and 82 dialogue sessions were conducted. We recorded these dialogue sessions with multi-modal sensors, including a 16-channel microphone array and HD cameras. The uttered speech was segmented by inter-pausal units (IPUs), with the segment for differentiation set at 200 milliseconds. This IPU setting is common in other studies on spoken dialogue systems (Skantze, 2021). Subjects’ audio data were enhanced by delay-and-sum beamforming (Ishi et al., 2016b). A shotgun microphone recorded the operator’s audio data.

### 3.2 Annotation

**FIGURE 3:**
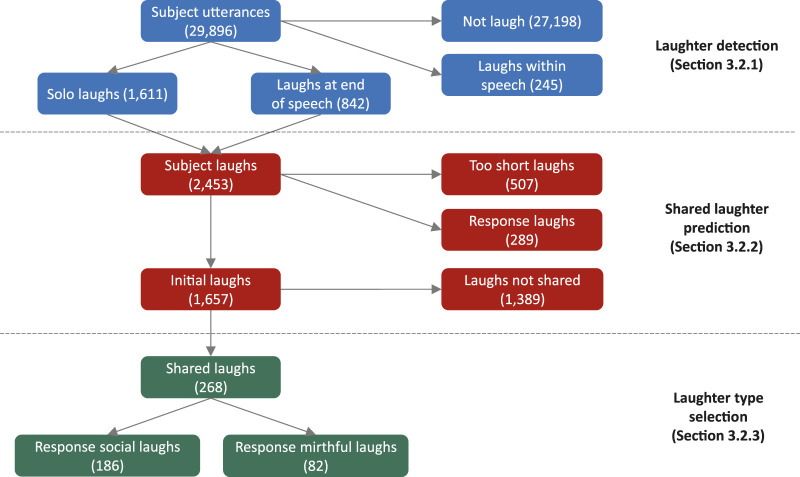
Diagram of annotated labels (number of extracted samples).

Using the speed-dating corpus, we conducted the following three annotation tasks to create shared laughter samples. Figure 3 summarizes the annotated labels and how many were extracted.

#### 3.2.1 Subject laughter

First, to train the laughter detection model, we annotated laughter samples uttered by the user in the speed dating dialogue corpus. The laughter samples were identified as “vocal laughs,” which exclude instances when users only show facial smiles. We included both “solo laughs” (i.e., “ha ha”) and “speech laughs” (speaking while laughing). In the current task, the proposed system is assumed to make a decision on shared laughter for each utterance instead of continuously. We only include samples uttered on the user’s turn because we want the system’s laughter to be a response to user dialogue. Furthermore, positive samples for speech laughs are the IPUs that end with laughter because we do not want the system to respond to laughs that were uttered too far into the past. This means that samples with a laugh at the beginning or middle of the utterance become negative samples. Consequently, we identified 2,453 IPUs (8.2%) as positive samples consisting of solo laughs (1,611) or speech laughs that ended with laughter (842) and 27,443 IPUs as negative samples consisting of non-laughs (27,198) and speech laughs that did not end with laughter (245).

#### 3.2.2 Shared laughter

Next, we used the positive samples extracted in the previous section for shared laughter annotation. We annotated the samples as shared laughter if ERICA laughed as a response to the initial user laugh in a timely manner. We first filtered out short and subtle laughs if the duration of the IPU containing a laugh was shorter than 400 milliseconds; 507 samples were filtered out.

We then determined a time-gap threshold for shared laughter to be 2 s, that is, if ERICA responded to a laugh sample with a laugh within 2 s after the end of the initial laugh, this sample was annotated as shared laughter. We encountered 289 cases of laughter for which the subject’s laughs were actually response laughs triggered by ERICA’s laughter. This situation mostly occurred when both participants uttered several laughs in sequence. We only considered the first triggering laughs in the sequence to be valid for model training and ignored the remainder of the sequence. In total, 268 shared laughter samples were extracted as positive samples of shared laughter for our model, whereas 1,389 were labeled as negative samples, indicating unshared laughter. Therefore, the accuracy of a random predictor based on this distribution is 16.2%.

#### 3.2.3 Laughter type

Finally, we annotated ERICA’s response laughs in the shared laughter interactions observed in the speed-dating dialogue corpus. Referring to several existing studies devoted to classifying laughter types, we decided to use a simple binary classification: mirthful and social (Tanaka and Campbell, 2011, 2014; Ishi et al., 2016a; Truong et al., 2019). Mirthful laughs are likely to be elicited by positive moods and expressed toward the dialogue itself, whereas social laughs tend to be used to augment and “fill” the conversation although humor is not involved. These include laughter that indicates embarrassment or shame, although we determined that few examples of these were in the corpus. In this study, we attempt to implement a model to select a mirthful or social laugh for the system’s response laugh based on the user’s initial laugh.

We used the 268 shared laughter samples from the speed-dating dialogue corpus and annotated each of ERICA’s response laughs as mirthful or social to be used as labels in the model. Five annotators listened to each laughing audio sample and selected its laughter type individually. It should be noted that they listened to only the laughing part, not the context or initial laugh, such that they focused on the target laughing sample. Before they began annotating, we also presented them with intelligible samples of each type as a reference. Although the boundary of the laughter types might depend on each annotator and these laughter samples were from actual dialogues not acted speech, the Fleiss’ kappa among the five annotators was 0.404, which is moderate agreement. Table 1 summarizes the distribution of the annotated labels. Finally, we took a majority vote for the final label, which resulted in 186 social and 82 mirthful laughs.

**TABLE 1:** Distribution of annotated samples on laughter type (Social: number of people who annotated the sample as social; Mirthful: number of people who annotated the sample as mirthful).

Social	Mirthful	#Sample
5	0	86
4	1	60
3	2	40
2	3	22
1	4	30
0	5	30

## 4 Proposed system

**FIGURE 4:**
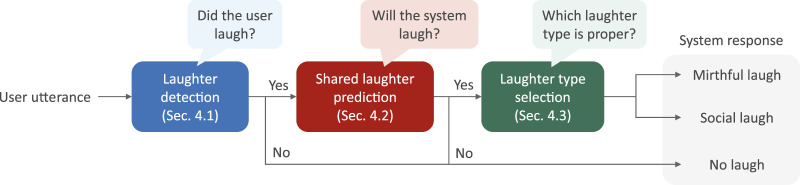
Architecture of the proposed system.

Figure 4 presents the architecture of the proposed system for shared laughter generation. The proposed system consists of three modules. First, when a user utterance segmented by pauses is input into the system, laughter is detected at the end of the utterance (Section 4.1). Second, if the utterance ends with laughter, whether the system also laughs with the initial user laugh is predicted (Section 4.2). Finally, if the system laughs, the laughter type (mirthful or social) is selected to be uttered by the system. This section explains each module and its evaluation.

### 4.1 Laughter detection

The first module of the proposed system is the user’s laughter detection. This classification is performed every time the system receives an IPU from the user.

#### 4.1.1 Model

The input is a user utterance segmented by pauses, and the features are a sequence of 40-dimensional Mel-filterbank coefficients denoted as $\left(x_{1},x_{2}\cdots ,x_{T_{i}}\right)$ , where *T*_*i*_ is the length of the sequence on the *i*-th user utterance. This feature is frequently used in other audio processing tasks, such as automatic speech recognition. The output is a binary value *y*_*i*_ corresponding to the probability of the utterance ending with laughter.

**FIGURE 5:**
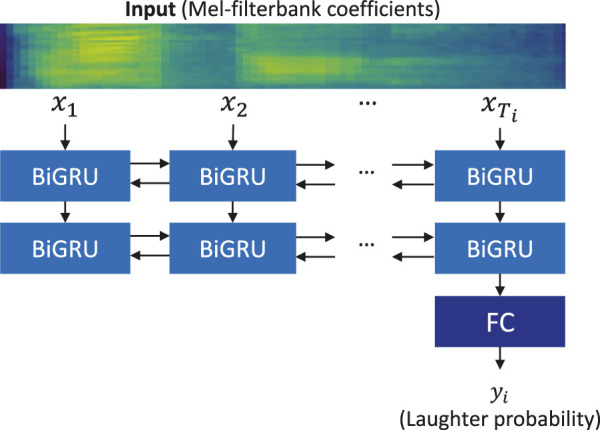
Recurrent neural network for laughter detection (FC: fully connected layer).

A recurrent neural network is applied to laughter detection, using the model architecture illustrated in Figure 5. First, an input sequence of the Mel-filterbank coefficients is fed to a stacked recurrent neural network. Then, the output of the last frame *T*_*i*_ is fed to the fully connected layer and soft-max function to obtain the laughter probability *y*_*i*_. The recurrent neural network is implemented using the bi-directional gated recurrent unit (BiGRU). Although this network is simple, the feed-forward processing can work in real time, which is required by the proposed system.

#### 4.1.2 Evaluation

We then evaluated the laughter detection model with the annotated data described in Section 3.2.1. The number of layers for the BiGRUs was 2, and the dimension of the hidden layers was 256. The dropout ratio for model training was 0.3. The Adam optimizer was used, where the learning rate was 1 × 10^−3^ and the weight decay was 1 × 10^−4^. Gradient clipping was applied with a threshold of 5.0. The number of training epochs was 50, and the mini-batch size was 64. A cross-entropy loss function was used.

The input features were z-score normalized with the mean and variance values calculated from the entire training dataset. The same normalization was performed for the test data with the mean and variance values calculated during training. A data augmentation approach, SpecAugment (Park et al., 2019), was applied to mask the time and frequency directions randomly. To ease the label imbalance, we conducted random up-sampling of the laughter samples. The evaluation metrics were the precision, recall, and F1 score of the laughter samples, and a 10-fold cross-validation was conducted.

Table 2 reports the results. Both the data augmentation and up-sampling were effective, and the F1 score reached 82.6%. Recent studies also reported similar F1 scores. For example, a ResNet model with a data augmentation achieved an F1 score of 75.2% in the switchboard telephone conversation corpus (Gillick et al., 2021). Therefore, our laughter detection module is expected to work with sufficient accuracy in the proposed system.

**TABLE 2:** Laughter detection performance (%).

	Precision	Recall	F1 score
BiGRU	82.4	75.8	79.0
+ SpecAugment	85.3	78.7	81.8
+ Up-sampling	78.2	87.6	82.6

### 4.2 Shared laughter prediction

The second module of the proposed system is shared laughter prediction. This task is triggered when the previous module detects a user’s laugh, and the prediction is made based on the features of this laugh. Shared laughs are expected to accompany large user laughs but not small chuckles, and we know from our corpus analysis that not responding with a laugh is more common.

#### 4.2.1 Model

The inputs of the shared laughter prediction model were the acoustic and prosodic features of the preceding user laugh. The acoustic features are the mean and variance values of the 40-dimensional Mel-filterbank coefficients, resulting in 80 dimensions. The prosodic features are the mean, median, standard deviation, maximum, minimum, and range values of fundamental frequency (F0) and power, resulting in 12 dimensions. It should be noted that the above feature set can be extracted in real time.

The prediction model was implemented with logistic regression. Although the current task has a limited amount of training data, using neural networks did not show any performance gains in our preliminary experiment, suggesting that a larger number of training samples is required for more sophisticated models. We also explored the linguistic features of preceding user utterances, such as bag-of-words and BERT-based embedding, but these did not contribute to the model improvement. Although linguistic features depend on automatic speech recognition accuracy and processing time, we decided to exclude them from the proposed system.

#### 4.2.2 Evaluation

The shared laughter prediction model was evaluated with the annotated data described in Section 3.2.2. The evaluation metrics were the precision, recall, and F1 score of shared laughter, and a 10-fold cross-validation was also conducted.

Table 3 reports the evaluation results. The acoustic features were more effective than the prosodic features. Although the scores were low because of the class imbalance and limited training data, using both feature sets achieved better performance than random prediction. Therefore, the acoustic and prosodic features of the initial laugh can provide some benefit for shared laughter prediction. This performance is also evaluated on the entire system in Section 5.

**TABLE 3:** Shared laughter prediction performance (%).

Feature	Precision	Recall	F1 score
Random	16.2	16.2	16.2
Acoustic (A)	20.3	52.2	29.2
Prosodic (P)	17.8	46.3	25.7
A + P	21.2	53.4	30.3

### 4.3 Laughter type selection

The third module of the proposed system is laughter type selection. This task is triggered when the previous module decides the system will perform shared laughter. Given the user’s initial laugh as input, the system predicts the laughter type to use for the response laugh.

We use the same logistic regression model as in the shared laughter prediction, with the input features being the acoustic and prosodic features calculated from the preceding user laugh and the output being the classification as a social or mirthful laugh.

#### 4.3.1 Evaluation

We evaluated the laughter type selection model with the annotated data described in Section 3.2.3. The evaluation metrics were the precision, recall, and F1 score of both mirthful and social laughs, and the macro F1 scores with 10-fold cross-validation. Table 4 reports the evaluation result. The results suggest that the prosodic features were more significant than the acoustic features. This result suggests that the proper laughter type (mirthful or social) relates to prosodic patterns of the initial laughs such as pitch and power. Intuitively, it is expected that stronger power and higher pitch induce mirthful laughs. Therefore, we decided to use only prosodic features in the proposed system for the full subjective evaluation described in the next section.

**TABLE 4:** Laughter type selection performance (%).

Feature	Mirthful laugh			Social laugh			Macro F1 score
	Precision	Recall	F1 score	Precision	Recall	F1 score	
Random	30.6	30.6	30.6	69.4	69.4	69.4	50.0
Acoustic (A)	48.5	61.0	54.1	80.6	71.5	75.8	64.9
Prosodic (P)	54.9	68.2	60.8	84.3	75.2	79.5	70.2
A + P	52.6	61.0	56.5	81.5	75.8	78.6	67.5

It is to be noted that although these models provide objective evaluations, we also require subjective evaluations of the system to obtain an idea of how the system would be perceived in a real interaction. Therefore, we conducted a subjective listening experiment.

## 5 Subjective evaluation

We integrated the three modules of the proposed system into a spoken dialogue system. Then, a subjective evaluation was conducted in which crowdsource workers evaluated the performance of the entire system.

### 5.1 Methodology

In this experiment, the proposed system was implemented using the attentive listening system of the android ERICA which generates listener responses, such as backchannels (Inoue et al., 2020). The purpose of the attentive listening system is to elicit user conversations by demonstrating understanding and empathy from the system. However, the conversation is primarily driven by the user. This means that the evaluation of the system is based on listening behavior instead of the dialogue content. Shared laughter is expected to contribute to this. The integrated system first identifies user utterances using real-time power-based voice activity detection. Then, it detects user laughs and utters system response laughs (or does not respond) based on the decisions of the proposed models. ERICA’s text-to-speech (TTS) system can provide natural recorded laughing utterances as opposed to synthesized laughs. We manually selected several mirthful and social TTS laughs. When the system decides on a laughter type, it then randomly picks one of them within the selected category.

We created four attentive listening scenarios in which a user talks for 2–3 min. The users were two males, including the first author. Table 5 summarizes the statistics of the four scenarios. In the first scenario, only social laughs were uttered, and only mirthful laughs were uttered in the second and third scenarios. In the fourth scenario, both types were uttered. These audio samples can be accessed on our website^1^.

**TABLE 5:** Distribution of annotated samples on laughter type (social: number of people who annotated the sample as social; mirthful: number of people who annotated the sample as mirthful).

Scenario	Speaker (user)	Length	#User laughs	#Shared laughter (#mirthful/#social)	#Evaluators
1	A	2 min 26 s	6	3 (0/3)	41
2	B	1 min 21 s	3	2 (2/0)	31
3	A	2 min 2 s	5	2 (2/0)	30
4	A	3 min 17 s	12	7 (4/3)	30

The talks with the integrated attentive listening system were recorded, and we then manually edited the responses of the system to create two comparable baselines. The first baseline, “no laugh,” corresponds to the existing attentive listening system that does not generate laughter. Instead of uttering response laughs, the listener responds with silence. The second baseline, “reactive,” is a system that always utters a social laugh when it detects a user laugh. This baseline system corresponds to the proposed system without shared laughter prediction or laughter type selection. This can be implemented using existing modules, such as laughter detection, and uses only social laughs, which are the majority in our corpus. This baseline system can be found in existing systems that can generate system laughs (Türker et al., 2017). It is a conservative laughing behavior strategy that minimizes the risk of making dialogue unnatural through excessive mirthful laughter.

We recruited more than 30 crowdsource workers to listen to each scenario with three different conditions and then evaluate each condition. Four metrics were evaluated: empathy, naturalness, human likeness, and understanding. The perceptions of these metrics are expected to be improved by shared laughter behaviors. Our previous experiment on the attentive listening system revealed that empathy and understating separate the system and human listeners (Inoue et al., 2020). Thus, these metrics should be addressed by additional behaviors, such as shared laughter. The evaluation sentences were presented to the workers as follows:

• How much did the female-voiced robot empathize with the male user? (empathy)
• How much were the responses of the female-voiced robot natural? (naturalness)
• How much was the female-voiced robot human-like? (human likeness)
• How much did the female-voiced robot understand what the male user was saying? (understanding)

These metrics were evaluated on a 7-point scale from 1 to 7, with 7 being the highest. Note that this experiment was conducted in the Japanese language.

### 5.2 Results and analysis

Table 6 summarizes the evaluation results. First, for the mean values, the proposed system scored the highest against the two baseline systems on all four metrics. Since the score trends seem to depend on each scenario, we conducted a paired one-sided *t*-test on each metric and scenario. We validated two comparisons: 1) the proposed system and the first baseline (no laugh) and 2) the proposed system and the second baseline (reactive). The *p*-values were modified according to the Holm correction.

**TABLE 6:** Mean values on evaluation scores in a subjective experiment (Emp: empathy, Nat: naturalness, Hum: human likeness, Und: understanding).

Scenario	Proposed				No laugh (baseline 1)				Reactive (baseline 2)			
	Emp	Nat	Hum	Und	Emp	Nat	Hum	Und	Emp	Nat	Hum	Und
1	4.54	3.88	4.12	4.24	4.39	3.98	3.83	4.20	4.00	3.20	3.37	3.66
2	4.39	3.48	3.87	4.10	3.97	3.42	3.81	3.58	4.29	3.68	4.23	4.13
3	4.30	3.77	4.07	4.30	4.10	3.87	3.97	3.90	4.93	4.03	4.60	4.57
4	5.23	4.97	5.47	5.00	4.70	4.30	4.43	3.93	5.10	4.63	4.73	4.83
Mean	4.61	4.01	4.36	4.39	4.30	3.89	3.99	3.92	4.53	3.83	4.16	4.24

In the first scenario (*n* = 41), the proposed system showed significantly higher scores than the second baseline (reactive) on all the metrics: empathy (*p* = 0.003), naturalness (*p* < 0.001), human likeness (*p* < 0.001), and understanding (*p* = 0.003). The difference between them was the timing of shared laughter because all the selected laughter types were social. Therefore, we can say that shared laughter must be used selectively in this scenario. On the other hand, there was no significant difference between the proposed method and the first baseline (no laugh).

In the second scenario (*n* = 31), there was a significant difference between the proposed system and the first baseline (no laugh) for understanding (*p* = 0.046). Since the length of this scenario is short and the number of laughs generated is fewer than those of the other scenarios, the difference in the evaluation is not so large. In other words, this scenario did not require many shared laughs, and the proposed system also works well in this kind of scenario, not degrading the quality of interaction.

In the third scenario (*n* = 30), the second baseline (reactive) showed significantly higher scores than the proposed system on empathy (*p* = 0.002) and human likeness (*p* = 0.037). This result suggests that shared laughter prediction failed in this scenario, and it also supports the importance of shared laughter prediction for improving the impression of the system and dialogue.

In the fourth scenario (*n* = 30), the proposed system showed significantly higher scores than the first baseline (no laugh) on all the metrics: empathy (*p* = 0.016), naturalness (*p* < 0.014), human likeness (*p* < 0.001), and understanding (*p* < 0.001). Compared to the second baseline (reactive), the proposed system showed significantly higher scores on naturalness (*p* = 0.030) and human likeness (*p* < 0.001). This scenario is the longest and observed the most user and shared laughs. The proposed system selectively uttered shared laughs and seemed to properly select mirthful and social laughs. In such a scenario, the proposed system achieved higher scores than the others. In summary, the abovementioned analyses suggest that the proper generation of shared laughter contributes favorably to the impression of the system in attentive listening dialogue across several scenarios with differing frequencies and types of shared laughter.

### 5.3 Discussion

We emphasize the use of social laughs. They compose a majority of the laughs in our corpus but are often subtle and quieter than mirthful laughs. Moreover, although we have the benefit of being able to select social laughs in ERICA’s system, other agent systems tend to have more explicit laughter (if any). Scenario 3 provides us with evidence that reactive social laughs are sufficient for a shared laughter system in some cases. We propose that social laughs, which are often overlooked, are an important feature of a dialogue system. With advances in laughter generation and synthesis, creating a variety of laughs for differing situations will be possible.

This work had several limitations. Collecting a large number of shared laughter samples from natural dialogue corpora is difficult. Most laughs are actually unshared laughs. A larger dataset is expected to be required for implementing richer features such as in linguistic and video data. Furthermore, attentive listening is user-initiated, so user laughs are more likely to be observed. The system utters only listener responses. Therefore, noticing differences in laughing behaviors among the conditions is easier. The differences may not be as apparent in a task with more mixed initiative. The effectiveness of shared laughter in other dialogue tasks must be validated.

## 6 Conclusion

We have presented a shared laughter generation system for a robot based on initial laughs provided by the user. The proposed system consists of three modules: initial laugh detection, shared laughter prediction, and laughter type selection. We evaluated each module with a speed-dating dialogue corpus. Laughter detection was implemented using a recurrent neural network, and the F1 score reached 82.6%. Shared laughter prediction and laughter type selection also showed higher scores than a random model. The proposed system was integrated into the spoken dialogue system of the android ERICA.

In the subjective evaluation, the proposed system was integrated into the attentive listening system. Dialogue audio samples were evaluated by crowdsource workers, and shared laughter generation was suggested to contribute to improving the impression of the system and dialogue in the attentive listening scenario. Our results suggest that the perception of shared laughter is influenced by the scenario and type of laugh used and emphasize the importance of proper empathetic laughs.

This study was conducted in the Japanese language and with a limited number of samples. However, the framework of the proposed method can be applied to other languages as well. Laughter is a non-linguistic behavior but is also dependent on the context of dialogue, including culture. Therefore, it is a future task to verify the generalizability of the proposed method in other languages and with large-scale data.

## Data Availability

The original contributions presented in the study are included in the article/Supplementary Material; further inquiries can be directed to the corresponding author.

## Footnotes

1
http://sap.ist.i.kyoto-u.ac.jp/members/inoue/slg/

## References

[B1] Akhtar Z. Bedoya S. Falk T. H. (2018). “Improved audio-visual laughter detection via multi-scale multi-resolution image texture features and classifier fusion,” in International Conference on Acoustics, Speech and Signal Processing (ICASSP), 3106–3110. 10.1109/ICASSP.2018.8461611

[B2] Annamoradnejad I. Zoghi G. (2020). ColBERT: Using BERT sentence embedding for humor detection. arXiv [Preprint]. 10.48550/arXiv.2004.12765

[B3] Ataollahi F. Suarez M. T. (2019). “Laughter classification using 3D convolutional neural networks,” in International Conference on Advances in Artificial Intelligence (ICAAI), 47–51. 10.1145/3369114.3369142

[B4] Batliner A. Steidl S. Eyben F. Schuller B. (2019). On laughter and speech-laugh, based on observations of child-robot interaction. arXiv [Preprint]. 10.48550/arXiv.1908.11593

[B5] Bonin F. Campbell N. Vogel C. (2014). Time for laughter. Knowledge-Based Syst. 71, 15–24. 10.1016/j.knosys.2014.04.031

[B6] Chen P.-Y. Soo V.-W. (2018). “Humor recognition using deep learning,” in Conference of the North American Chapter of the Association for Computational Linguistics: Human Language Technologies (NAACL-HLT), 113–117. 10.18653/v1/N18-2018

[B7] Cosentino S. Sessa S. Takanishi A. (2016). Quantitative laughter detection, measurement, and classification–a critical survey. IEEE Rev. Biomed. Eng. 9, 148–162. 10.1109/rbme.2016.2527638 26887012

[B8] El Haddad K. Chakravarthula S. N. Kennedy J. (2019). “Smile and laugh dynamics in naturalistic dyadic interactions: Intensity levels, sequences and roles,” in International Conference on Multimodal Interaction (ICMI), 259–263. 10.1145/3340555.3353764

[B9] Estow S. Jamieson J. P. Yates J. R. (2007). Self-monitoring and mimicry of positive and negative social behaviors. J. Res. Personality 41, 425–433. 10.1016/j.jrp.2006.05.003

[B10] Gillick J. Deng W. Ryokai K. Bamman D. (2021). Robust laughter detection in noisy environments. Interspeech, 2481–2485. 10.21437/Interspeech.2021-353

[B11] Glenn P. J. (1991). Current speaker initiation of two-party shared laughter. Res. Lang. Soc. Interact. 25, 139–162. 10.1080/08351819109389360

[B12] Glenn P. (2003). Laughter in interaction. Cambridge: Cambridge University Press. 10.1017/CBO9780511519888

[B13] Gosztolya G. Tóth L. (2019). Calibrating DNN posterior probability estimates of HMM/DNN models to improve social signal detection from audio data. Interspeech, 515–519. 10.21437/Interspeech.2019-2552

[B14] Gupta R. Chaspari T. Georgiou P. G. Atkins D. C. Narayanan S. S. (2015). Analysis and modeling of the role of laughter in motivational interviewing based psychotherapy conversations. Interspeech, 1962–1966. 10.21437/Interspeech.2015-432

[B15] Hara K. Inoue K. Takanashi K. Kawahara T. (2018). Prediction of turn-taking using multitask learning with prediction of backchannels and fillers. Interspeech, 991–995. 10.21437/Interspeech.2018-1442

[B16] Hofmann J. Platt T. Ruch W. Niewiadomski R. Urbain J. (2015). The influence of a virtual companion on amusement when watching funny films. Motiv. Emot. 39, 434–447. 10.1007/s11031-014-9461-y

[B17] Holt E. (2010). The last laugh: Shared laughter and topic termination. J. Pragmat. 42, 1513–1525. 10.1016/j.pragma.2010.01.011

[B18] Hussain N. Erzin E. Sezgin T. M. Yemez Y. (2019). Speech driven backchannel generation using deep Q-network for enhancing engagement in human-robot interaction. arXiv [Preprint]. 10.48550/arXiv.1908.01618

[B19] Inoue K. Lala D. Yamamoto K. Nakamura S. Takanashi K. Kawahara T. (2020). “An attentive listening system with android ERICA: Comparison of autonomous and WOZ interactions,” in Annual Meeting of the Special Interest Group on Discourse and Dialogue (SIGDIAL), 118–127.

[B20] Inoue K. Milhorat P. Lala D. Zhao T. Kawahara T. (2016). “Talking with ERICA, an autonomous android,” in Annual Meeting of the Special Interest Group on Discourse and Dialogue (SIGDIAL), 212–215. 10.18653/v1/W16-3625

[B21] Ishi C. Hatano H. Ishiguro H. (2016a). “Audiovisual analysis of relations between laughter types and laughter motions,” in Speech Prosody, 806–810. 10.21437/SpeechProsody.2016-165

[B22] Ishi C. Liu C. Even J. Hagita N. (2016b). “Hearing support system using environment sensor network,” in International Conference on Intelligent Robots and Systems (IROS), 1275–1280. 10.1109/IROS.2016.7759211

[B23] Ishi C. T. Minato T. Ishiguro H. (2019). Analysis and generation of laughter motions, and evaluation in an android robot. APSIPA Trans. Signal Inf. Process. 8, 1–10. 10.1017/atsip.2018.32

[B24] Kantharaju R. B. Ringeval F. Besacier L. (2018). “Automatic recognition of affective laughter in spontaneous dyadic interactions from audiovisual signals,” in International Conference on Multimodal Interaction (ICMI), 220–228. 10.1145/3242969.3243012

[B25] Lala D. Inoue K. Kawahara T. (2020). “Prediction of shared laughter for human-robot dialogue,” in Companion Publication of International Conference on Multimodal Interaction (ICMI), 62–66. 10.1145/3395035.3425265

[B26] Lala D. Nakamura S. Kawahara T. (2019). Analysis of effect and timing of fillers in natural turn-taking. Interspeech, 4175–4179. 10.21437/Interspeech.2019-1527

[B27] Luong H.-T. Yamagishi J. (2021). Laughnet: Synthesizing laughter utterances from waveform silhouettes and a single laughter example. arXiv [Preprint]. 10.48550/arXiv.2110.04946

[B28] Mori H. Nagata T. Arimoto Y. (2019). Conversational and social laughter synthesis with wavenet. Interspeech, 520–523. 10.21437/Interspeech.2019-2131

[B29] Navarretta C. (2016). “Mirroring facial expressions and emotions in dyadic conversations,” in International Conference on Language Resources and Evaluation (LREC), 469–474.

[B30] Neves L. Cordeiro C. Scott S. K. Castro S. L. Lima C. F. (2018). High emotional contagion and empathy are associated with enhanced detection of emotional authenticity in laughter. Q. J. Exp. Psychol. 71, 2355–2363. 10.1177/1747021817741800 PMC6261327 30362411

[B31] Niewiadomski R. Hofmann J. Urbain J. Platt T. Wagner J. Piot B. (2013). “Laugh-aware virtual agent and its impact on user amusement,” in International Conference on Autonomous Agents and Multi-agent Systems (AAMAS), 619–626.

[B32] Niewiadomski R. Pelachaud C. (2012). “Towards multimodal expression of laughter,” in International Conference on Intelligent Virtual Agents (IVA), 231–244. 10.1007/978-3-642-33197-8_24

[B33] Park D. S. Chan W. Zhang Y. Chiu C.-C. Zoph B. Cubuk E. D. (2019). SpecAugment: A simple data augmentation method for automatic speech recognition. Interspeech, 2613–2617. 10.21437/Interspeech.2019-2680

[B34] Provine R. R. (1992). Contagious laughter: Laughter is a sufficient stimulus for laughs and smiles. Bull. Psychon. Soc. 30, 1–4. 10.3758/bf03330380

[B35] Provine R. R. (2001). Laughter: A scientific investigation. London: Penguin.

[B36] Skantze G. (2021). Turn-taking in conversational systems and human-robot interaction: A review. Comput. Speech & Lang. 67, 101178101178–101178101226. 10.1016/j.csl.2020.101178

[B37] Tanaka H. Campbell N. (2011). “Acoustic features of four types of laughter in natural conversational speech,” in International Congress of Phonetic Sciences (ICPhS), 1958–1961.

[B38] Tanaka H. Campbell N. (2014). Classification of social laughter in natural conversational speech. Comput. Speech & Lang. 28, 314–325. 10.1016/j.csl.2013.07.004

[B39] Tits N. Haddad K. E. Dutoit T. (2020). Laughter synthesis: Combining seq2seq modeling with transfer learning. arXiv [Preprint]. 10.48550/arXiv.2008.09483

[B40] Truong K. P. Trouvain J. (2014). Investigating prosodic relations between initiating and responding laughs. Interspeech, 1811–1815. 10.21437/Interspeech.2014-412

[B41] Truong K. P. Trouvain J. Jansen M.-P. (2019). Towards an annotation scheme for complex laughter in speech corpora. Interspeech, 529–533. 10.21437/Interspeech.2019-1557

[B42] Truong K. P. Van Leeuwen D. A. (2007). Automatic discrimination between laughter and speech. Speech Commun. 49, 144–158. 10.1016/j.specom.2007.01.001

[B43] Türker B. B. Buçinca Z. Erzin E. Yemez Y. Sezgin T. M. (2017). Analysis of engagement and user experience with a laughter responsive social robot. Interspeech, 844–848. 10.21437/Interspeech.2017-1395

[B44] Turker B. B. Yemez Y. Sezgin T. M. Erzin E. (2017). Audio-facial laughter detection in naturalistic dyadic conversations. IEEE Trans. Affect. Comput. 8, 534–545. 10.1109/taffc.2017.2754256

[B45] Urbain J. Bevacqua E. Dutoit T. Moinet A. Niewiadomski R. Pelachaud C. (2009). “AVLaughterCycle: An audiovisual laughing machine,” in International Summer Workshop on Multimodal Interfaces, 79–87.

[B46] Weller O. Seppi K. (2019). “Humor detection: A transformer gets the last laugh, ” in Conference on Empirical Methods in Natural Language Processing and International Joint Conference on Natural Language Processing (EMNLP-IJCNLP), 3621–3625. 10.18653/v1/D19-1372

[B47] Yalçın O. N. DiPaola S. (2019). “Evaluating levels of emotional contagion with an embodied conversational agent,” in Aannual conference of the cognitive science society (CogSci), 3143–3149.

